# Roles of Insulin Receptor Substrates (IRS) in renal function and renal hemodynamics

**DOI:** 10.1371/journal.pone.0242332

**Published:** 2020-12-03

**Authors:** Seiji Hashimoto, Tomochika Maoka, Tetsuya Kawata, Toshio Mochizuki, Takao Koike, Takashi Shigematsu

**Affiliations:** 1 Department of Nephrology, Kinan Hospital, Tanabe, Wakayama Prefecture, Japan; 2 NTT East Japan Sapporo Hospital Department of Nephrology, Chuou-ku, Sapporo, Japan; 3 Hokkaido University Graduate School of Medicine, Internal Medicine II, Kita-ku, Sapporo, Japan; 4 Department of Nephrology, Wakayama Medical University, Wakayama, Wakayama Prefecture, Japan; University Medical Center Utrecht, NETHERLANDS

## Abstract

We have reported previously that renal hemodynamic abnormalities exist in the prediabetic stage of type II diabetic rats. At this prediabetic stage these rats have hyperinsulinemia, insulin resistance and metabolic syndrome. It is well known that insulin resistance is frequently associated with renal abnormalities, but the mechanism underlying this association has remained speculative. Although insulin is known to modify renal hemodynamics, little is known about the roles of insulin receptor substrates (IRS1, IRS2) in the renal actions of insulin. To address this issue, the effects of insulin on renal function and renal hemodynamics were investigated in C57BL/6 (WT: wild type), insulin receptor substrate 1- knockout (*IRS1–/–*), and IRS2-knockout (*IRS2–/–*) mice. IRS2–/–mice had elevated glucose level as expected. 24-h urine collections and serum creatinine revealed that creatinine clearance did not significantly differ between these groups. Albuminuria was found in *IRS1–/–*and *IRS2–/–*groups. We examined the effects on the IRS during the administration of Losartan, which is widely used for diabetic nephropathy. After the administration of Losartan the IRS displayed improved renal hemodynamics. Moreover, the subjects were also given Pioglitazone, which improves insulin resistance. Losartan significantly reduced albuminuria in both groups. Pioglitazone also showed similar results. We assessed the autoregulatory responses of the total renal blood flow (RBF), the superficial (SBF) and the deep renal cortical blood flow (DBF) with stepwise reductions of renal perfusion pressure (RPP), which was induced by a manual clamp on the abdominal aorta. During the clamp induced reductions of the RPP by 10 to 20mm HG, RBF, SBF and the DBF fell significantly more in the IRS1 and IRS2 than in the WT mice. Furthermore micropuncture studies showded that compared to the WT tubuloglomerular feedback (TGF) responses of the stop flow pressure (P_sf_) were reduced in both the *IRS1 -/-* and *IRS2 -/-*. The results of the IRS1 and IRS2 mice displayed the pressence of hemodynamic abnormalities. Losartan and Pioglitazone have shown the potential to improve these abnormalities. In conclusion the results indicate that IRS plays a major role in the stimulation of renal functions and renal hemodynamics in type type II diabetes.

## Introduction

Chronic kidney disease (CKD) is a worldwide confronted problem in that approximately 20% of overall population is affected by this disorder. Particularly, in developed countries, diabetes mellitus is the primary factor for initiating dialysis therapy and measures are being sought to control the disease [1, 2]. Insulin, needless to mention, is a hormone that centrally functions to control the energy storage/supply system of the body. Failure of the insulin function system, referred to as insulin resistance, is an element of prime importance that characterizes diabetes mellitus. The energy metabolism-regulating effect of insulin is exerted via insulin receptors and is mediated by the IRS-PI3-kinase pathway [3]. With the progress in elucidation of these receptors in recent years, proteins such as insulin receptor substrates (IRS1 and IRS2) have been identified and, currently [4, 5], mice with a genetically deleted IRS are prepared in laboratories [6, 7]. Evidence has been accumulating to corroborate involvement of IRS1 primarily in the insulin effects in skeletal muscles and that of IRS2 in the hepatic insulin effects.

Meanwhile, the presence of insulin abnormalities in patients with renal failure has been known for a relatively long time [8]. In 1983, this was clinically verified by DeFronzo et al. using the clamping technique [9]. Thus, it is generally recognized that insulin resistance exists from a relatively early stage of CKD.

Conversely, there is a report of a large-scale clinical trial demonstrating that insulin resistance *per se* constitutes a risk factor for progression of CKD. Iseki et al. reported that elevated BMI represents a risk of disease progression into terminal renal failure in male patients [10], and that the ARIC and NHANEIII Studies showed metabolic syndrome to be a risk factor for the development of CKD [11, 12].

In CKD, insulin resistance arises depending on the type of disease state and insulin resistance associated with renal failure itself worsens CKD, so that there is the possibility of a vicious cycle occurring between the two. It has also been pointed out that insulin resistance may have a bearing on CDK-associated cardiovascular disease/cardio-renal association [13]. However, the relationship between nephropathies and insulin resistance remains unclear in many respects, and reports particularly dealing with relationship with insulin receptors are as yet sparse. This report describes our study on the relationship between insulin receptors and nephropathies.

## Methods

All studies were approved by and performed in compliance with the guidelines and practices of Hokkaido University Graduate School of Medicine.

All animal studies and procedures were approved by Hokkaido University Animal Research Committee (permit no. H21-0106).

### (1) Animals

The following two types of genetically modified mice obtained from the laboratory of Dr. T. Kadowaki, the University of Tokyo, were used: IRS1 knockout mice (*IRS1****-/-****)* [6] and IRS2 knockout mice (*IRS2****-/-***) [14]. These two types of knockout mice had been derived by genetic engineering from the background strain of C57/BL6(WT: wild Type) as previously described. They were accommodated in thermostat-controlled rearing cages (Hokudo Co., Ltd., Sapporo) at the Laboratory Animal Care and Use Facility, Hokkaido University. The animals were supplied with tap water ad libitum and allowed free access to certified mouse diet (MF^®^; Oriental Yeast Co., Ltd., Tokyo). They were housed ≤5/cage in the rearing cages maintained at a constant temperature (24°C) and relative humidity (40–60%) in a stream of sterilized air. After the end of the experiment, mice were euthanized by intravenous overdose of pentobarbital according to the guidelines for euthanasia of experimental animals.

### (2) Urine collection test

A 24-hour urine was collected from each female mouse aged between 8 and 12 weeks by means of a metabolism cage under conditions of free access to drinking water and diet. SRL Inc. (Tokyo, Japan) was entrusted with determination of urinary creatinine concentration (enzymatic method) in the 24-hour urine collection. Urinary albumin concentration was determined using Mouse Albumin ELISA Kit (AKRAL-121, Shibayagi, Gunma, Japan). After the collection of 24-hour urine specimens, the animals were sacrificed and blood samples were drawn. Blood glucose levels were determined by using a GR102 (TERMO, Tokyo, Japan), and serum samples were assayed for urea nitrogen (UN; urease colorimetry), creatinine (Cr; enzymatic method) and protein (Biuret method). SRL Inc. was also entrusted also with determination of urinary creatinine concentration (enzymatic method) and urine assay for protein (colorimetry) upon sacrifice of the animals.

### (3) Animal preparation

Renal hemodynamic measurements were done according to the report of Hashimoto et al. [15, 16]. Namely animals were anesthetized with thiobutabarbital (Inactin^®^;Research Biochem-icals Incorporated, Natick, MA, USA, 100 mg/kg i.p.), and ketamine (Ketalar^®^; Daiichsankyou Co Ltd, Tokyo, Japan, 100 mg/kg i.m.). Body temperature was maintained at 38°C by placing the animals on an operating table with a servo-controlled heating plate. The trachea was cannulated, and a stream of 100% oxygen was blown towards the tracheal tube throughout the experiment. The femoral artery was cannulated with hand-drawn polyethylene tubing for continuous measurement of arterial blood pressure (AP-601G, Nihon Kohdn Co Ltd, Tokyo, Japan) and blood withdrawal. The jugular vein was cannulated for an intravenous maintenance infusion of saline at 0.35 ml/hr. A catheter was placed in the bladder for urine collections.

#### Measurements of total RBF and superficial renal blood flow

Renal blood flow (RBF) was measured. The left renal artery was approached from a flank incision and carefully dissected free to permit placement of a 0.5-mm V-type ultrasonic flow probe (Transonic Systems, Ithaca, N.Y., USA). In the candesartan series, a 0.5PSB nanoprobe with a T402-PB flowmeter was used (Transonic Systems). The probes were held in place with a micromanipulator. Mean arterial pressure monitored in the lower abdominal aorta was regarded as renal perfusion pressure (RPP). RPP was set to the desired level by a manual clamp placed above the branching sites of both renal arteries. RPP was reduced in three stages by tightening the clamp mildly or more severely. The left renal artery was approached from a flank incision and carefully dissected free to permit placement of a Doppler blood flow transducer (internal diameter, 1.0 mm; HDP-10, Crystal Biotech Northborough, MA, USA) connected to a 20-MHz module (PD-20, Crystal Biotech) and dedicated amplifier (VF-1, Crystal Biotech). Regional blood flow of the left kidney was monitored with two glass fiber probes connected to a real-time dual laser Doppler flow meter (PeriFlux System 5000; Perimed Inc., Stockholm, Sweden). For recordings of superficial and deep cortical flow signals the probes were held in place at the surface and at a depth of about 1 mm respectively, and regarded to register superficial (SBF) and deep cortical flow (DBF). RPP, SBF and DBF signals were digitized and analyzed using MacLab software (AD Instruments, Colorado Springs, CO, USA). These methods were also done in the same way as previously reported [17].

### (4) Losartan and pioglitazone dosing experiment

Both *IRS1****-/-*** and *IRS2****-/-*** mice were administered losartan, an angiotensin receptor inhibitor, or pioglitazone, a drug for improvement in insulin resistance, and their responses were observed.

Both these knockout mice approximately 8- to 12-weeks of age and wild type mice of the same age were subjected to 24-hour urine collection in the manner described in (2) above. The both knockout mice and WT mice were allocated respectively to 2 groups to receive losartan or pioglitazone. An Alzet Osmotic Pump Model 2004 was implanted in the dorsal back of each mouse under anesthesia with intraperitoneal thiobutabarbital at 100 mg/kg. Mice of the losartan group were administered a solution of 9 mg losartan in 0.2 mL of water per day for 28 days via the implanted pump. Likewise, mice of the pioglitazone group received a solution of 13 mg pioglitazone in 0.2 mL of water per day for 28 days via the pump.

On Day 25 after the start of dosing, a 24-hour urine test was carried out in the same manner under the conditions of free access to drinking water and diet. Urine samples collected were assayed for creatinine and albumin.

### (5) Micropuncture studies

Micropuncture studies were done according to the report of Hashimoto et al. [15, 16]. The left kidney was approached from a flank incision, freed from fat and tissue connections, and placed in a Lucite cup. Stop-flow pressure (P_sf_) as an index of glomerular capillary pressure was determined during loop perfusions at 0 and 30 nl/min as described previously. The sequence of the flow change was randomized. The following perfusion fluid was used (in mM): 136 NaCl, 4 NaHCO3, 4 KCl, 2 CaCl2, 7.5 urea, and 100 mg/100 ml FD&C green (Keystone Scientific, Bellefonte, Pa., USA).

### (6) Statistical analysis

Data were expressed as mean ± S.D. Inter-individual comparison and comparison of intraglomerular pressure between the superficial and deep layers of the mouse kidney were performed using analysis of variance (ANOVA). The level of significance was set at P < 0.05, and the statistical analysis was carried out using Excel Statistics 2006 (SSRI Co., Ltd., Tokyo).

## Results

### (1) Basic tests and urine collection test

Body weight data, 24-hour urine test data, and blood biochemical test data for the *IRS1****-/-***, *IRS2****-/-***, and WT mice are summarized in Table 1 (WT: n = 9, *IRS1****-/-***: n = 11, *IRS2****-/-***: n = 9).

**Table 1:** Functional parameters in mice.

	C57BL/6 Mice	*IRS1 -/-* Mice	*IRS2 -/-* Mice
N (animals)	9	11	9
Body Weight (g)	26.6 ± 2.1	16.2 ± 2.2*	32.3 ± 1.8*
s Cr (mg/dL)	0.11 ± 0.01	0.06 ± 0.02*	0.11 ± 0.04
BUN (mg/dL)	30.2 ± 6.8	23.7 ± 3.8*	29.4 ± 5.2
s Na (mmol/L)	151 ± 2.4	150 ± 5.0	149 ± 1.8
BG (mg/dL)	158 ± 35	185 ± 37	270 ± 44*
UV (ml/day)	1.5 ± 0.7	1.1± 0.8	2.1± 0.8*
Urine Cr (μg/day)	342 ± 93	182 ± 96*	343 ± 94
Ccr (μl/min)	226 ± 75	209 ± 112	226 ± 49
Ccr (μl/min/g)	0.9 ± 0.3	1.3 ± 0.8	0.7 ± 0.1

s Cr: serum Creatinine, BUN: Blood Urea Nitrogen, s Na: serum sodium, BG: Blood Glucose, UV: Urine Volume, Urine Cr: Urine Creatinine, Ccr: Creatinine clearance

Values are means S.D.

* p < 0.05: vs C57/BL6-

Body weights of *IRS1****-/-*** mice were significantly lower as compared with WT mice, whereas *IRS2****-/-*** mice showed significantly greater body weights than the WT mice. The serum creatinine (Cr) level was significantly lower in the *IRS1****-/-*** mice, compared to the WT mice and did not significantly differ between the *IRS2****-/-*** mice and the WT mice. The same trend was noted with respect to serum blood urea nitrogen (BUN) level, being significantly lower in the *IRS1****-/-*** mice than in the WT mice and showing no significant difference between the *IRS2****-/-*** mice and the WT mice. The plasma glucose level (PG) was significantly higher in the *IRS2****-/-*** mice than in the WT mice, whilst there was no significant difference in this respect between the *IRS1****-/-*** mice and the WT mice. The urine output was significantly greater in the *IRS2****-/-*** mice and was somewhat smaller though without significant difference in the IRS1**-/-** mice, as compared with the WT mice. The *IRS1****-/-*** mice exhibited a significant depression of urinary Cr excretion as compared with that in theWT mice, while the urinary Cr excretion did not significantly differ between the *IRS2****-/-*** mice and the WT mice. The creatinine clearance (Ccr) calculated from urinary Cr excretion and urine volume did not significantly differ between any two groups of mice.

### (2) Measurement of renal hemodynamics

After securing time control data following baseline determinations in a steady state, the renal perfusion pressure (RPP) was reduced in uniform decrements of approximately 10 mmHg to mild clamp (E1) and further to moderate clamp (E2) by manual clamping of the abdominal aorta in each mouse to continuously record the renal blood flow (RBF), superficial renal cortical blood flow (SBF), and deep renal cortical blood flow (DBF) at those clamping time points. Stable data obtained from 6 *IRS1****-/-*** mice, 8 *IRS2-/-* mice, and 6 WT mice were adopted for analysis.

**Fig 1:**
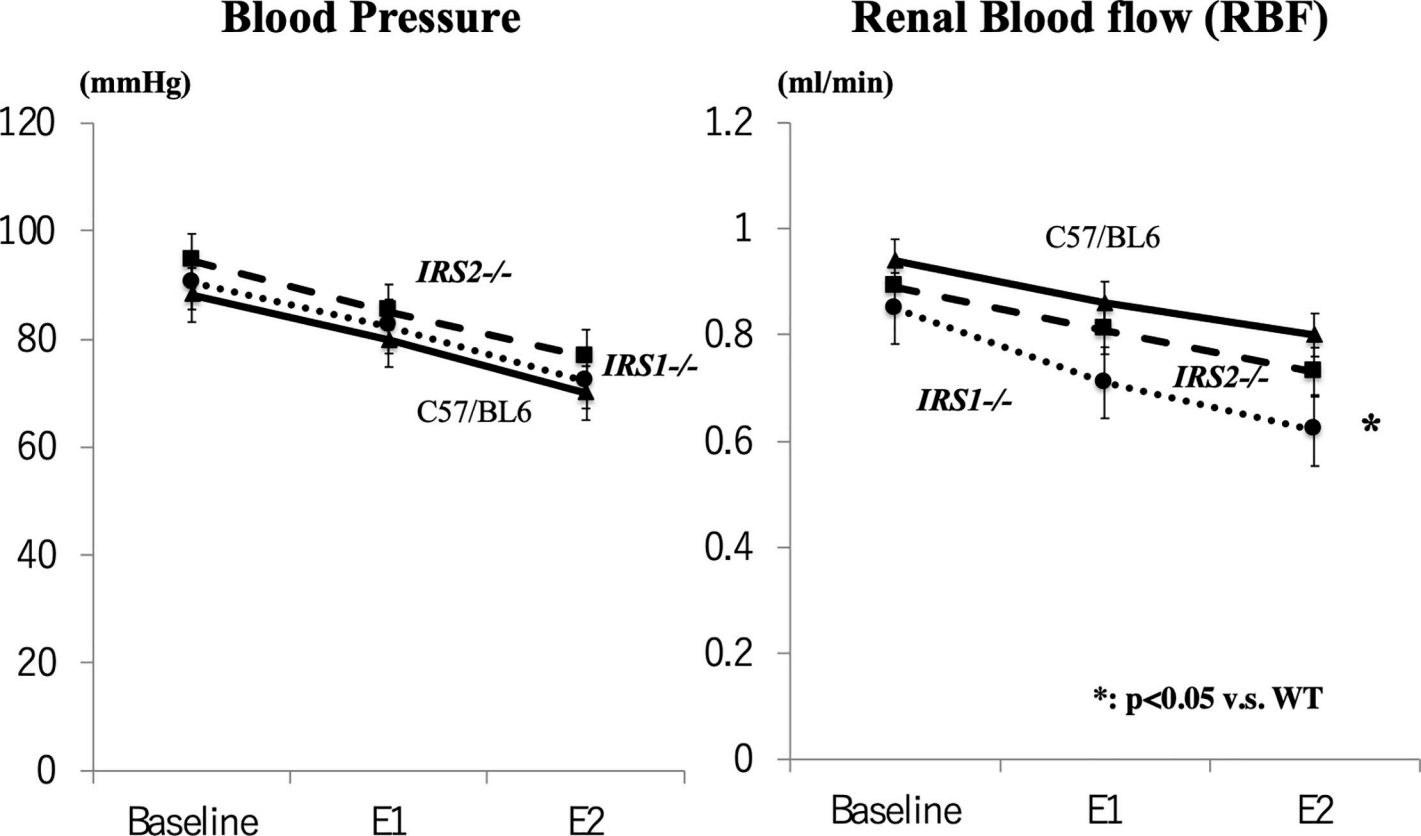
Measurement of renal hemodynamics. Blood Pressure (Left) and Renal Blood Flow (RBF) (Right). Baseline: before study (control), E1: mild clamp, E2: severe clamp, Bar: WT, dotted line: *IRS1-/-*, broken line: *IRS2-/-*.

Fig 1 depicts changes observed in RPP. There was no appreciable difference in baseline blood pressure among the *IRS1-/-* mice (90.5 ± 5.5 mmHg), *IRS2-/-* mice (94.5 ± 3.3 mmHg), and WT mice (88.2 ± 2.0 mmHg). The blood pressure in terms of RPP was lowered to E1 and further to E2 almost equally in these 3 groups.

Changes observed in RBF on RPP modifications are also illustrated in Fig 1. The baseline RBF did not significantly differ among the *IRS1****-/-*** mice (0.85 ± 0.04 mL/min), *IRS2****-/-*** mice (0.89 ± 0.03 mL/min), and WT mice (0.94 ± 0.04 mL/min), yet tended to be slightly depressed in the *IRS1****-/****-* mice. There was no significant intergroup difference in RBF at E1 with an RPP reduced by approximately 10 mmHg. However, at E2 with an RPP reduced by approximately 20 mmHg from the baseline level, RBF was markedly lower in the IRS1-/- mice (0.62±0.03 mmHg) than in the WT mice.

**Fig 2:**
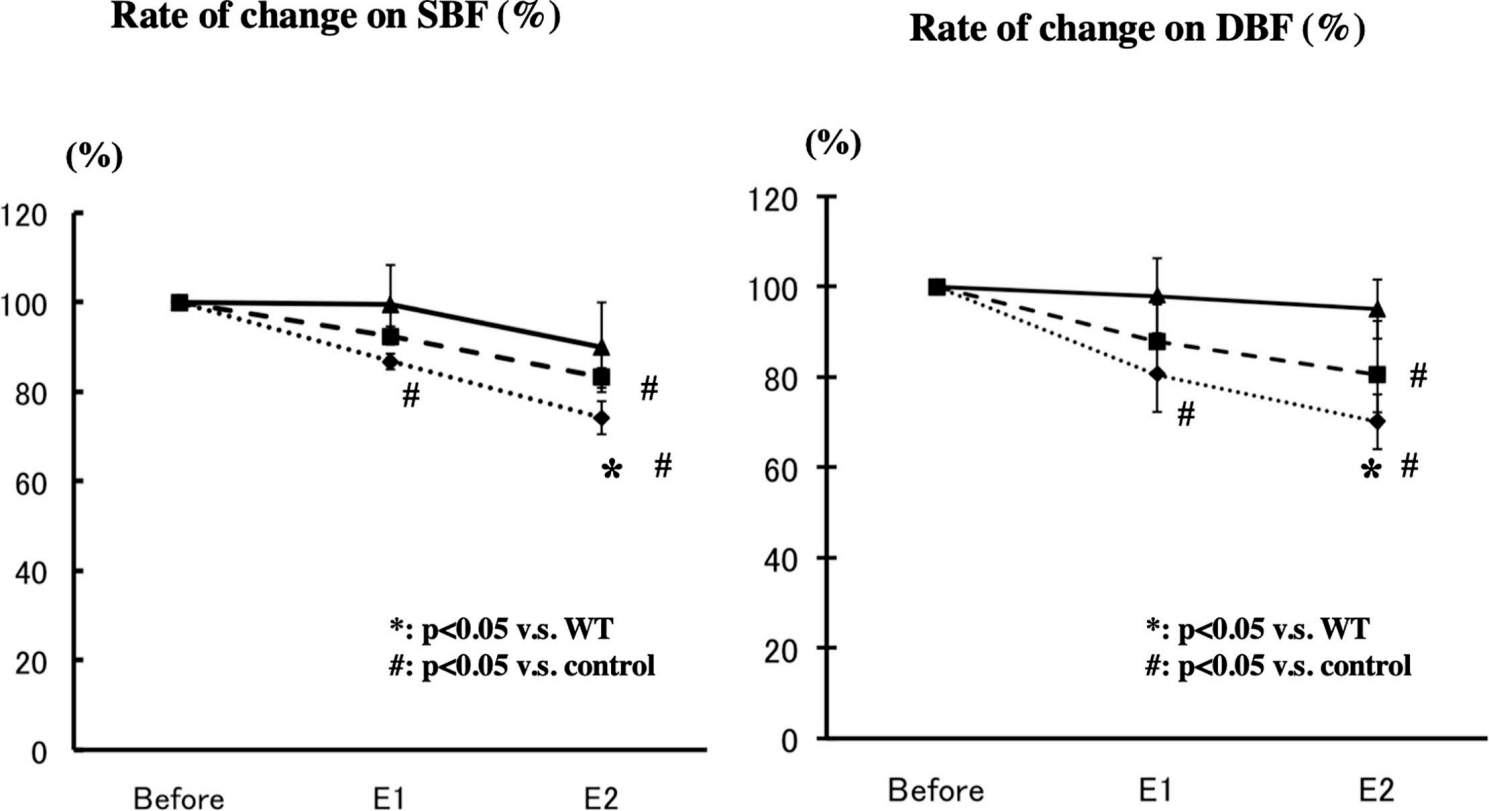
Superficial (SBF) and the deep renal cortical blood flow (DBF) with stepwise reductions of Renal Perfusion Pressure (RPP). SBF (Left) and DBF (RBF) (Right). Baseline: before study (control), E1: mild clamp, E2: severe clamp, Bar: WT, dotted line: *IRS1-/-*, broken line: *IRS2-/-*. *: p<0.05 v.s. WT, #: p<0.05 v.s. control.

Results of concurrent SBF and DBF determinations are presented in Fig 2, where the SBF and DBF are shown as percent of respctive baseline values. SBF tended to decrease at both E1 and E2 in *IRS1****-/-*** mice as well as in *IRS2-/-* mice, being significantly reduced (86%) in *IRS1****-/-*** mice, as compared with the WT mice (96%). At E2, the SBF was significantly reduced in the *IRS1****-/-*** mice (74%) and *IRS2****-/-*** mice (83%), compared with theWT mice mice (90%). The DBF similarly tended to decrease, with a significant decrease in *IRS1****-/-*** mice **(**81%) at E1 as compared with the WT mice (98%). At E2, the DBF was significantly reduced in the *IRS1****-/-*** *mice* (70%) and *IRS2****-/-*** *mice* (81%), compared with the WT mice (95%).

### (3) Changes in tubuloglomerular feedback mechanism

**Fig 3:**
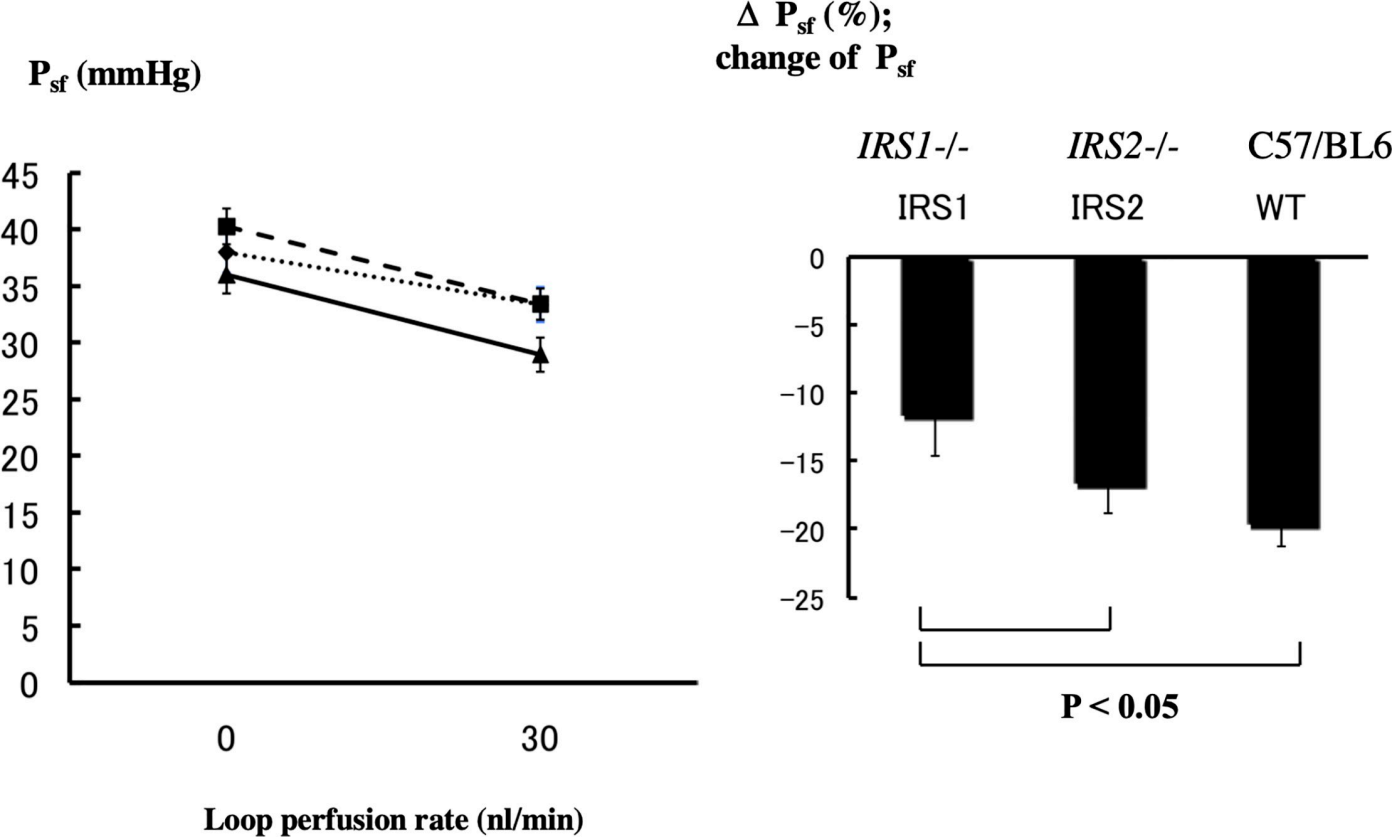
Micropuncture studies.

The mean stop flow pressure (P_sf_) measured at renal tubular perfusate flow rates of 0 and 30 nl/min into distal tubules and changes in mean P_sf_ as percent of value at 0 nl/min in the mouse groups are shown in Fig 3. The measurement was performed on 10 nephrons in the *IRS1****-/-*** mice, 14 nephrons in the *IRS2****-/-*** mice, and 12 nephrons in the WT mice.

The P_sf0_ in a steady state (0 nl/min) did not significantly differ among the *IRS1****-/-*** mice (38.0 ± 1.8 mmHg), *IRS2****-/-*** mice (40.3 ± 1.6 mmHg), and WT mice (36.0 ± 1.7 mmHg). At a renal tubular perfusate flow rate of 30 nl/min, there was a significant decrease in P_sf30_ in all groups, i.e., *IRS1****-/-*** (33.4 ± 1.6 mmHg), *IRS2****-/-*** (33.4 ± 1.4 mmHg), and WT mice (29.0 ± 1.7 mmHg).

When assessed in terms of rate of change in P_sf_ at 30 nl/min (⊿P_sf_) as against the steady state value, there were significantly smaller percentages of decrease in P_sf30_ in the *IRS1****-/-*** mice (−11.7 ± 2.9%) and *IRS2****-/-*** mice (−16.7 ± 2.0%), compared to the WT mice (−19.7 ± 1.5%).

### (4) Results of losartan and pioglitazone dosing experiment

**Fig 4:**
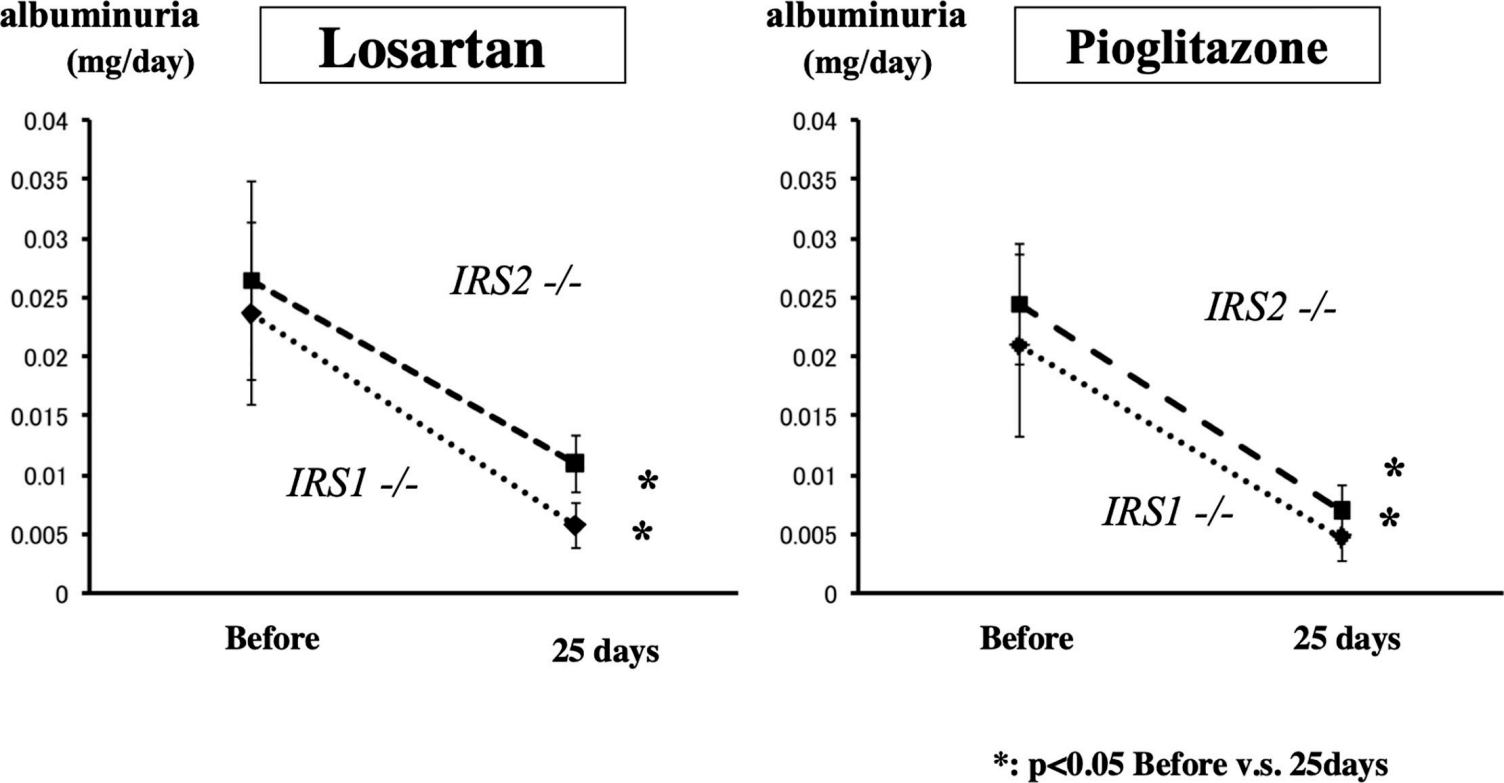
Results of administration of losartan and pioglitazone. Before: before study (control), 25 days: after continuous administration.

Results of tests on 24-hour urine specimens prior to the start of treatment with the drugs in both knockout (*IRS1****-/-*** and *IRS2-/-*) mouse groups and the WT mice group are presented in Fig 4. Albuminuria was significantly more frequent in both knockout (*IRS1****-/-*** and *IRS2-/-*) mouse groups than in the WT mice, while there was no significant difference between the *IRS1****-/-*** and *IRS2-/-* mouse groups in this respect. Both the IRS1**-/-** and *IRS2-/-* mouse groups exhibited a significant decrease in albuminuria in response to treatment with losartan (*IRS1****-/-***: from a pre-treatment value of 2.19 μg to 0.70 μg; and *IRS2****-/-***: from 2.54 μg to 1.23 μg). Both knockout mouse groups also showed a significant decrease in urine albumin/urine Cr ratio (*IRS1****-/-***: from a pre-treatment value of 0.13 to 0.05; and *IRS2****-/-***: from 0.13 to 0.05); hence, demonstrating an inhibitory effect of losartan on albuminuria. As was the case with losartan, both the *IRS1****-/-*** and *IRS2-/-* mouse groups displayed a significant decrease in albuminuria as well in response to treatment with pioglitazone (*IRS1****-/-***: from a pre-treatment value of 2.09 μg to 0.46 μg; and *IRS2****-/-***: from 2.44 μg to 0.69 μg).

## Discussion

It has become clarified in recent years that the essential nature of diabetes mellitus consists in insulin resistance, and insulin-sensitizing agents have been marketed in succession as drugs for treatment of insulin resistance. Basic elucidation of the disorder has been progressing to demonstrate that insulin binds to insulin receptor to activate its tyrosine kinase with ensuing tyrosine-phosphorylation of intracellular insulin receptor substrates such as IRS1 to 4 and shc. SH2 protein etc. are also bound to these substrates to exert insulin effects such as glucose uptake, protein synthesis and cell differentiation [18]. Thus, IRS proteins play an important role in the exertion of effects of insulin, and mice with a genetically deleted IRS are prepared for the elucidation of this mechanism.

IRS1**-/**- mice have growth retardation with a physique limited to about 2/3 of that of WT mice. They are hyperinsulinemic, showing blood glucose levels about twice as high as those in WT mice [6]. Though showing insulin resistance, they nevertheless have blood glucose levels confined within the normal reference range. In IRS2**-/-** mice, on the other hand, elevation of blood glucose emerges when they become about 10-week old, along with marked glucose intolerance. They show blood glucose levels about three times as high as those in WT mice. Obesity is evident in IRS2-knockout mice. It has been reported that the organ responsible for insulin resistance in IRS2**-/-** mice is the liver [14].

As for the relationship of the kidney with insulin, insulin metabolism and excretion become delayed with progressing renal failure. Eventually, depression of insulin clearance occurs in patients with renal insufficiency [19]. It is generally recognized that gluconeogenesis in the kidney accounts for 20% of total gluconeogenesis, and gluconeogenesis diminishes in association with depression of renal function [20]. Thus, it is relatively well known that improvement in blood glucose level occurs in patients with renal failure.

Hyperinsulinemia has a bearing on hypertension via its effects on the sympathetic nervous system and effects of sodium. Insulin has an effect of activating eNOS, so that it exerts a vasodilatatory action by enhancing NO production [21]. It has been reported that insulin produces an increase in renal blood flow, where vasodilatatory response of the afferent arterioles exceeds that of the efferent arterioles, consequently leading to elevation of filtration fraction (FF) [22, 23]. Increase in FF is thought to inversely correlate with insulin resistance, and may eventually give rise to glomerular hypertension and glomerular hyperfiltration [23]. It has long been known that insulin affects the renal hemodynamic state, and studies in recent years have demonstrated NOS-mediated influence of insulin on renal hemodynamics [24]. However, much remains unclear regarding the role played by IRS in the kidney. The present hemodynamic data analysis has shown failure in renal autoregulatory function at lowered perfusion pressure in the both IRS-knockout mice, suggesting possible involvement of IRSs in hemodynamic abnormalities. The abnormalities were noted to be more pronounced in the IRS1-knockout mice. Our previous report described that hemodynamic abnormalities are more conspicuous in the deep layer than in the superficial layer of the kidney in diabetic nephropathy [17]. The present data suggest the possibility of these abnormalities being IRS-mediated. It cannot be ruled out, nevertheless, that the changes in blood pressure etc. might possibly appear more pronounced in the IRS1**-/-** mice which were smaller in physique as compared with the other groups of mice.

Past reports have documented depression of tubuloglomerular feedback mechanism in diabetes mellitus [25]. The present study demonstrates depressed tubuloglomerular feedback in both IRS-knockout mice with no significant difference in this respect between the IRS1-knockout mice and the IRS2-knockout mice. It may be pointed out that insulin resistance and IRSs are likely to be involved in the depression of tubuloglomerular feedback mechanism diabetes mellitus.

Microalbuminuria was also noted in both the IRS1-knockout mice and IRS2-knockout mice. Reports concerning relationship of IRS with microalbuminuria are few as yet, but there are reports of human studies suggesting involvement of IRS1 [26]. From the present data, direct connection of insulin resistance or IRS abnormality with kidney dysfunction may be pointed out. Renal hemodynamic abnormality may be cited as a factor accountable for it.

For a long time, there have been reports of studies indicating that insulin and insulin resistance give rise to renal dysfunction while, conversely, impairment of kidney function brings about insulin resistance [27]. Taken together, it would be possible to infer that the events run in vicious cycles. Further, hyperinsulinemia accelerates the vicious cycle in terms of hypertension via its effects on the sympathetic nervous system and effects of sodium [28]. Both losartan and pioglitazone have proven to reduce microalbuminuria. Pioglitazone is an insulin-sensitizing agent, whose effects on IRS1 and IRS2 have been reported [29, 30]. It has been suggested that pioglitazone may produce improvement in insulin resistance and ameliorate albuminuria via IRS. However, it cannot be ruled out that the actions of drugs on the renal tubules themselves might have improved microalbumin levels. It has also been reported that pioglitazone affects the proximal tubules to alter albumin reabsorption [31, 32]. Further studies are needed. Furthermore, we did not assess the degrees of activation and inhibition of PPARγ and angiotensin receptors in our study. Thus, the extent of drug action is unknown.

Losartan, an inhibitor of rennin-angiotensin system (angiotensin receptor blocker; RAS), proved to be effective in both the IRS1-knockout mice and IRS2-knockout mice. Studies dealing with relationship of insulin resistance with the RAS have in recent years been published in succession. Angiotensin II (AngII) disturbs insulin signaling at various levels, thereby giving rise to insulin resistance [33]. AngII stimulates serine-phosphorylation of IRS1 in glucose-internalizing organs such as skeletal muscles. It has been reported that ARB has an effect of inhibiting the glucose uptake by such organs [34]. In the liver being a principal site for IRS2 abnormality, AngII causes hepatic fibrosis to exert insulin resistance [35]. Further, AngII has been reported to lower plasma adiponectin level [36]. It may be pointed out that ARB may improve insulin-sensitivity through these mechanisms. In our present study, losartan proved to reduce albuminuria in the IRS1-knockout mice with marked skeletal muscle abnormality as well as in the IRS2-knockout mice with marked hepatic abnormality. Although numerous studies have focused on the association of albuminuria with IRS or RAS, no conclusive evidence has been found. For example, because obesity-related nephropathy is well-known to be associated with proteinuria, obesity and Syndrome X appear to be one cause of proteinuria. However, IRS1-knockout mice are rather lean. Probably, the involvement of multiple factors, rather than a single factor, in the occurrence of proteinuria is highly likely.

It is universally recognized that losartan dilates efferent arterioles to improve renal hemodynamics with consequent lowering of intraglomerular pressure, thereby exerting a nephroprotective effect. Although there have been numerous reports about the effects of losartan and other ARBs on renal hemodynamics, few reports have focused on the effects of pioglitazone or PPARγ agonists on hemodynamics. However, there are some reports describing that PPARγ affects vascular smooth muscle or the RAS system. Investigating their effects on hemodynamics appears to be important [37].

It is evident from the present study and past reports that hemodynamic abnormalities exist in the presence of insulin resistance, and losartan is considered to exert its microalbuminuria-reducing effect by correcting the hemodynamic abnormalities.

## Conclusions

Nephropathies including abnormality in renal hemodynamics arise from IRS abnormalities. Losartan and pioglitazone are effective in improving those IRS-mediated abnormalities.

## Supporting information

S1 Data Alubuminuria. (XLS)

S2 Data Basic date. (XLSX)

S3 Data Blood flow. (XLS)

S4 Data Micropuncture IRS. (XLS)

S5 Data Renal blood flow. (XLS)
